# Biochemical Characterization of R-Loop Degradation by Chloroplast-Localized RNase H1 from *Arabidopsis thaliana*

**DOI:** 10.3390/ijms262211125

**Published:** 2025-11-17

**Authors:** Anastasia A. Gavrilova, Aleksandra A. Kuznetsova, Darya S. Novopashina, Chengxia Zheng, Qianwen Sun, Nikita A. Kuznetsov

**Affiliations:** 1 Institute of Chemical Biology and Fundamental Medicine, Siberian Branch of Russian Academy of Sciences, Novosibirsk 630090, Russia; anaroslakova@gmail.com (A.A.G.); danov@niboch.nsc.ru (D.S.N.); 2 Center for Plant Biology, School of Life Sciences, Tsinghua University, Beijing 100084, China; zheng-cx19@mails.tsinghua.edu.cn (C.Z.); sunqianwen@mail.tsinghua.edu.cn (Q.S.); 3 Department of Natural Sciences, Novosibirsk State University, Novosibirsk 630090, Russia

**Keywords:** enzyme kinetics, RNase H1, R-loop resolution, *Arabidopsis thaliana*, chloroplast, organelle genome stability

## Abstract

R-loops are three-stranded nucleic acid structures implicated in genome regulation and stability. In *Arabidopsis thaliana*, the chloroplast-localized RNase H1 enzyme (AtRNH1C) is important for chloroplast development and genome integrity; however, its molecular activity has not been experimentally verified. In the present study, we characterized the enzymatic activity of recombinant AtRNH1C toward model R-loops of various structures. Using a set of synthetic R-loop substrates, we demonstrate that AtRNH1C cleaves the RNA within DNA/RNA hybrids with a strong preference for purine-rich sequences, most notably at G↓X dinucleotides. Kinetic assays showed that the enzyme’s efficiency is highly dependent on the length of the hybrid duplex but is not affected by a G-quadruplex structure in the single-stranded DNA flap of the R-loop. The most rapid degradation was observed for an R-loop with an 11 nt DNA/RNA hybrid region. This study provides a comparative analysis of chloroplast-localized RNase H1 activity and elucidates its substrate preferences, suggesting that an R-loop with a heteroduplex length closest to the native size found in transcription elongation complexes is the most efficient substrate. These findings suggest that the enzymatic activity of AtRNH1C is sufficient to perform its function in maintaining chloroplast genome stability by the degradation of R-loops in DNA.

## 1. Introduction

R-loops are three-stranded nucleic acid structures formed by a DNA/RNA hybrid and a displaced single-stranded DNA. Research using the single-stranded DNA ligation-based library preparation method has revealed that R-loops are abundant in the *Arabidopsis* genome, particularly in gene promoters, gene bodies, and regions with GC/AT skews. These structures are also frequently found in repetitive regions, transposable elements, and noncoding RNAs, indicating their involvement in chromatin dynamics, gene expression, and plant-specific silencing pathways like RNA-directed DNA methylation [1,2]. In recent years, R-loops associated with transcription have been identified as a significant contributor to genome instability [3,4,5,6,7]. However, studies have shown that R-loops form independently of replication and that those formed in one cell cycle stage can persist into subsequent stages and be transmitted to the next generation [3,8,9]. Thus, R-loops are present throughout the cell cycle and influence a wide range of physiological and pathological processes [3,10,11]. The beneficial functions of R-loops encompass immunoglobulin class switch recombination, the regulation of gene expression, DNA replication, DNA repair, and the control of transcription initiation and termination [12,13]. Nevertheless, the aberrant accumulation of R-loops in the genome, whether in timing or location, exerts detrimental effects. These include transcriptional defects, transcription–replication conflicts, cell cycle arrest, and genomic instability [10,14,15,16]. This occurs because R-loops can block replication fork progression by provoking transcription–replication conflicts or by recruiting specific endonucleases that cause DNA cleavage. Consequently, cells have evolved multiple mechanisms to maintain R-loop homeostasis, which involve preventing R-loop formation, resolving existing R-loops, or repairing the resulting DNA damage such as fork blocks or DNA breaks [17,18,19,20].

Maintenance of R-loop homeostasis depends on a delicate balance between their formation and resolution. The proper regulation of R-loops necessitates a multitude of factors, given their genomic prevalence and broad functional influence. Essential to this control is a dedicated network of enzymes that modulates R-loop levels [18,19,20,21,22]. Helicases, enzymes that unwind nucleic acids, can prevent the formation of secondary structures [23]. Interactome analysis of RNA/DNA hybrids has revealed that many helicases, in addition to other proteins—including topoisomerases, RNA processing factors, and chromatin proteins—function as potential R-loop regulators [24,25,26,27]. Rho-n domain-containing protein 1 (RHON1) is the only identified RNA/DNA helicase in plants known to resolve R-loops caused by head-on transcription–replication conflicts in the *Arabidopsis* chloroplast genome [2,28]. By relaxing supercoiled DNA to relieve topological stress, DNA topoisomerases play a key role in replication and transcription. The absence of Topoisomerase I (TOP1) in human cells results in context-dependent alterations to R-loop levels, characterized by an increase at long, highly transcribed genes and a decrease at early replication origin-associated loci [29]. A conserved role is observed in rice, where TOP1 is indispensable for preventing R-loop accumulation on auxin-related genes to ensure proper root development [2]. Chromatin condensation, which influences transcription and replication states, is also associated with genome-wide R-loop formation [30]. This condensation is modulated by various histone modifications and the deposition of histone variants, processes carried out by chromatin remodelers [31]. Although chromatin remodelers are strongly associated with R-loop formation in vivo and recent studies have connected them to R-loops [30], direct evidence of their role in R-loop modulation or their mechanistic contribution to R-loop regulation remains scarce. Few plant chromatin remodelers have been directly connected with different stress responses [32,33].

The RNase H enzyme family, which includes RNase H1 and RNase H2, is evolutionarily conserved and specializes in degrading the RNA component of DNA/RNA hybrids [34], making these enzymes key players in R-loop resolution [35,36]. In *A. thaliana*, three genes encode RNase H1-like proteins: RNH1A (nuclear), RNH1B (mitochondrial), and RNH1C (chloroplast) [2,37]. The mitochondrial RNase H1, AtRNH1B, plays a critical role in maintaining R-loop homeostasis to preserve mitochondrial genome integrity and support early embryogenesis [2]. Specifically, AtRNH1B prevents genome instability by suppressing ectopic homologous recombination at intermediate-sized repeats in mitochondrial DNA. When AtRNH1B is absent, the chloroplast-localized RNase H1, AtRNH1C, relocates to mitochondria, compensating for the loss and ensuring genome stability. In chloroplasts, which possess their own genome [38], nucleoids are associated with thylakoid membranes [39]—a location that makes them vulnerable to environmental fluctuations, including DNA-damaging agents produced during photosynthesis [40,41]. Studies indicate that AtRNH1C is essential for regulating R-loop levels to maintain chloroplast genome stability, a process that requires collaboration with DNA gyrases [2]. Double mutants lacking both AtRNH1B and AtRNH1C demonstrated embryonic lethality due to R-loop accumulation, impaired mitochondrial DNA replication, and mitochondrial dysfunction, highlighting a facultative dual targeting mechanism between organelles [2].

**Figure 1:**
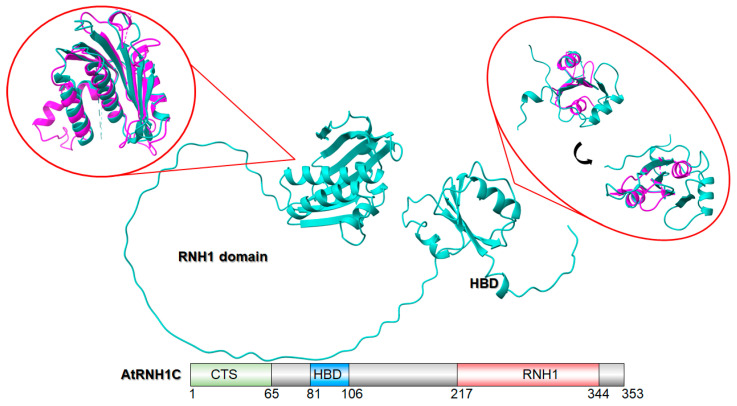
AlphaFold-predicted structure of AtRNH1C (AF–F4I7R5–F1–model_v4) and its domain composition. Red circles include the superposition of AtRNH1C and HBD domains with those of human RNase H1 (human RNH1—PDB ID 2KQ9; human HBD—PDB ID 3BSU). CTS, chloroplast targeting sequence (green on domain composition panel); HBD, hybrid binding domain (blue on domain composition panel); RNH1, RNase H1 domain (red on domain composition panel). The predicted structure represents the mature protein after the CTS has been cleaved.

RNase H1s are ubiquitous enzymes found in nearly all living organisms. Their catalytic domain, spanning approximately 150 amino acids (aa), is evolutionarily conserved [42]. In eukaryotes, RNase H1 contains an N-terminal mitochondrial targeting sequence (MTS) [43] and an additional ∼50 aa region known as the hybrid binding domain (HBD) [44]. The HBD is linked to the C-terminal catalytic domain by a flexible linker, which is essential for RNase H1 activity [45]. This structural arrangement confers significant flexibility, enabling the N- and C-terminal domains to reposition relative to one another. Figure 1 displays the AlphaFold-predicted structure of AtRNH1C. When superimposed with human RNase H1 (highlighted in red circles in Figure 1, the RNH1 domains show high structural similarity, while the HBD domains exhibit only partial convergence. The HBD is critical for the processive cleavage pattern unique to eukaryotic RNase H1. In yeast, mouse, and human enzymes, deletion of the HBD abolishes processivity, resulting in distributive cleavage of long DNA/RNA substrates—similar to the behavior of *E. coli* RNase H1, which naturally lacks an HBD [46]. A proposed model suggests that processive RNA cleavage involves multiple hydrolysis events triggered by a single HBD-mediated binding event. This mechanism generates short RNA fragments that readily dissociate from the DNA template [47]. Such processive cleavage is essential for efficiently degrading long substrates.

While RNase H1′s role in R-loop resolution and genome stability is well-documented in eukaryotic nuclei, the molecular functions of organelle-localized RNase H1 remain poorly understood. In *A. thaliana*, nuclear AtRNH1A and mitochondrial AtRNH1B are dispensable for development under standard laboratory conditions [37], whereas AtRNH1C is essential for chloroplast development [48]. However, its enzymatic activity has yet to be verified experimentally. This work directly addresses this gap by moving beyond genetic evidence to provide the first direct in vitro biochemical analysis of AtRNH1C. We characterize the enzymatic activity of recombinant AtRNH1C toward model R-loops in vitro. It was shown that AtRNH1C exhibits a strong preference for short R-loop structures (11 bp), which mirrors the natural length of hybrids found in transcription elongation complexes. The kinetic and mechanistic analysis reveals that AtRNH1C operates via a two-step mechanism common to bacterial RNase H1, where the rate-limiting step is the formation of the catalytic complex. The calculated rate constant is remarkably similar to that of *E. coli* RNase H1, underscoring a deeply conserved evolutionary strategy for R-loop resolution across billions of years of evolution.

## 2. Results

### 2.1. Design of R-Loops

**Figure 2:**
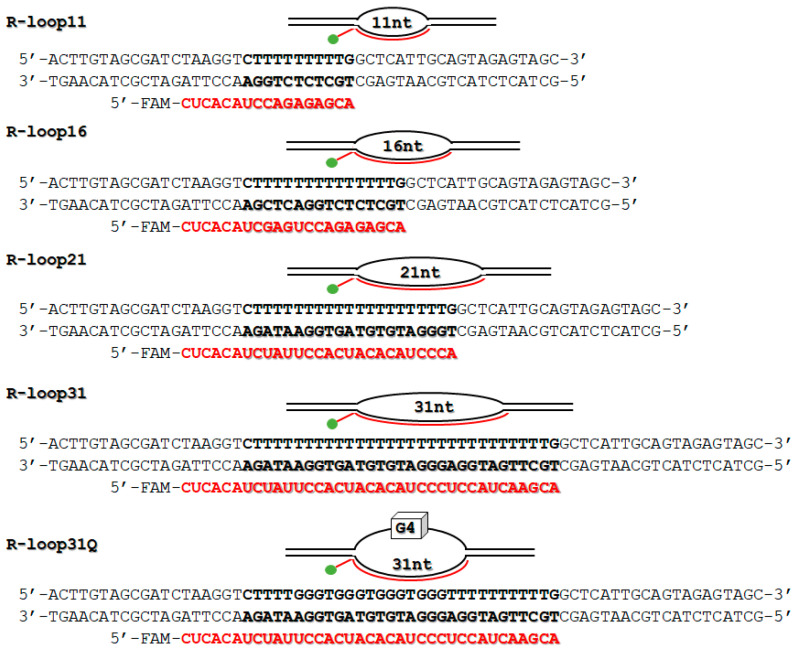
Oligonucleotide sequences and model R-loop structures. DNA strands colored black, RNA-primer colored red. Bold sequence denotes the DNA/RNA heteroduplex region. 6-carboxyfluorescein (FAM, green circles) was attached to the 5′ end of the RNA-primer.

In transcription elongation complexes, a specialized wedge-like structure in RNA polymerase limits the DNA/RNA hybrid to approximately 10–11 base pairs (bp) [49]. Therefore, the R-loop structure with 11 bp heteroduplex length (R-loop11) was used as a “natural” model (Figure 2). To systematically evaluate the influence of hybrid length, extended variants—R-loop16, R-loop21, and R-loop31—were also utilized. All oligonucleotide sequences were deliberately designed to ensure stable R-loop formation and to avoid intramolecular hairpins. Furthermore, the R-loop31Q variant was incorporated, featuring a G-quadruplex (G4) motif in the displaced non-template DNA strand, to probe the effects of DNA secondary structure on AtRNH1C function. This is relevant as G-rich sequences can form stable G4 structures that contribute to R-loop stability and are implicated in regulating genes for stress response and DNA damage [50,51,52]. The R-loops’ assembly was confirmed using EMSA as previously described [53].

### 2.2. Sequence Preferences of Purified AtRNH1C

**Figure 3:**
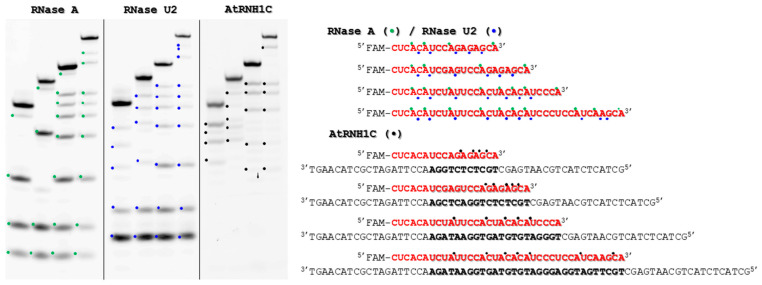
Analysis of AtRNH1C cleavage of DNA/RNA heteroduplexes. RNases A and U2 were used as control enzymes. The digestion of RNA (for RNase A and U2) or DNA/RNA heteroduplexes (for AtRNH1C) was performed as described in Section 4. For each substrate, the RNA sequences are shown. RNA chains are colored in red, DNA chains are colored in black and heteroduplex moieties are highlighted in bold. The circles indicate the sites of the enzymes digestion: green for RNase A, blue for RNase U2, and black for AtRNH1C.

Site and sequence preferences for cleavage by AtRNH1C were identified using R-loops (Figure 2) with different lengths and nucleotide sequences of the hybrid region. The RNases A and U2 were used as control enzymes. The RNA cleavage occurred at the hybrid regions at Pu↓X sites with preferred cleavage at a G↓X dinucleotide (Figure 3).

The obtained data demonstrated the AtRNH1C preference for a purine-rich sequence context. It is interesting to note that the efficient cleavage of the RNA strand in the sequence context CA↓AG, with a preference for the G-rich sequence upstream of the cleavage site, has been shown for *E. coli* RNase H1 and the catalytic domain of human RNase H1 [54].

### 2.3. R-Loop Degradation Activity

**Figure 4:**
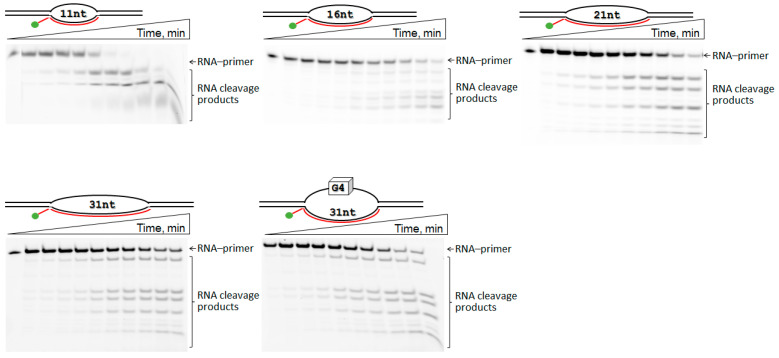
PAAG analysis of AtRNH1C-driven cleavage of the RNA in model R-loops. [AtRNH1C] = 2 µM, [R-loop] = 1 µM. Time: 0, 1, 2, 3, 5, 10, 15, 20, 30, 45, and 60 min. RNA and DNA chains are highlighted in red and black, respectively.

**Figure 5:**
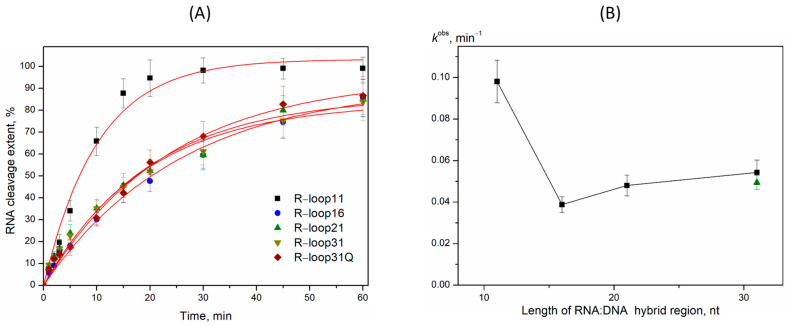
Kinetics of RNA cleavage by AtRNH1C in model R-loops. (**A**) Time-dependent cleavage of the RNA strand. The RNA cleavage extent was calculated as the ratio of the summed peak areas for all cleavage fragments to the total peak area (fragments and initial substrate). (**B**) Observed rate constants (kobs) derived from the kinetic curves in (**A**). The data for R-loop31Q is denoted by a triangle (▲). All data points represent the mean ± SD of three independent experiments.

A kinetic analysis of RNA primer cleavage by AtRNH1C in various R-loops was performed (Figure 4). As shown in the polyacrylamide gels, the full-length RNA primer disappeared over time, with a corresponding accumulation of multiple shorter product bands. For instance, in the R-loop11 substrate, the full-length RNA band was completely depleted within 15–20 min. The accumulation of these shortened RNA products is expected to destabilize the DNA/RNA hybrid, leading to R-loop resolution. The resulting kinetic curves for the cleavage reaction were well fitted by a single-exponential equation, allowing for the determination of the observed rate constant (*k*^obs^). The values of observed rate constant for R-loop16–31 were decreased with increasing hybrid region length (Figure 5). Moreover, R-loop11 showed the highest *k*^obs^ value among the substrates investigated (Figure 5). Recently, we performed the investigation of the kinetic features of the interaction of RNase H1 from *Escherichia coli* (EcRNH1) with similar model R-loops [53]. It was also found that EcRNH1 bound most effectively to R-loops with a 10–11 nt DNA/RNA hybrid region. The rate-limiting step in RNA degradation by EcRNH1 was identified as the formation of the catalytic complex. We therefore propose that the lower cleavage efficiency of AtRNH1C toward the larger R-loop16–31 is caused by both a higher number of potential “landing sites” in the longer heteroduplex and less efficient formation of the catalytic complex.

**Scheme 1:**
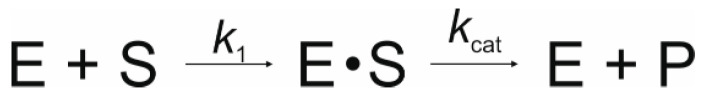
The kinetic scheme of the AtRNH1C-driven cleavage of the R-loop. E: AtRNH1C; S: an R-loop; E•S: the catalytic complex of the R-loop with the enzyme; P: a reaction product.

**Figure 6:**
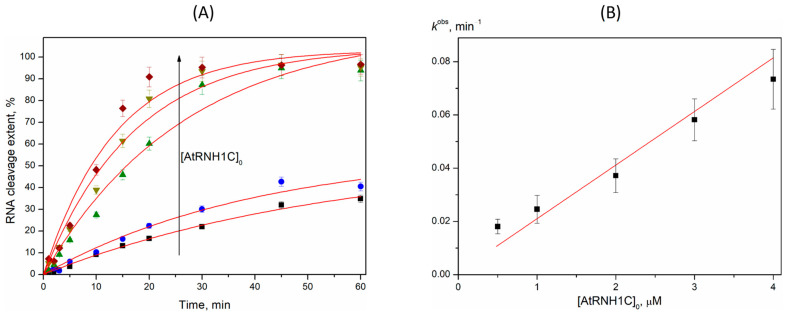
Enzyme concentration dependence of R-loop11 cleavage by AtRNH1C. (**A**) Progress curves of RNA cleavage at varying enzyme concentrations (0.5–4 µM). The RNA cleavage extent was calculated as the total area of product peaks divided by the sum of the product and initial substrate peak areas. Symbol traces represent experimental data (AtRNH1C, µM: ■ 0.5; ● 1; ▲ 2; ∇ 3; ♦ 4); red curves are the results of fitting to a single-exponential function. Values are the mean of three independent experiments. (**B**) Linear dependence of the observed rate constant (*k*^obs^) on AtRNH1C concentration.

A kinetic analysis of R-loop11 cleavage was conducted using 1 µM substrate and AtRNH1C concentrations between 0.5 and 4 µM (Figure 6). The cleavage time courses fit well to a single-exponential function, from which the observed rate constant (*k*^obs^) was derived for each enzyme concentration. A linear relationship was observed between *k*^obs^ and the initial AtRNH1C concentration. These data support that the kinetic scheme of AtRNH1C action could be described by a two-step mechanism (Scheme 1) if the rate constant of the catalytic reaction *k*_cat_ satisfies the condition *k*_cat_ >> S × *k*_1_. The same dependence of RNA cleavage was previously reported for R-loop degradation by EcRNH1 [53]. A linear dependence of *k*^obs^ on enzyme concentration allows us to calculate an apparent second-order rate constant (*k*_1_), which is effectively *k*_cat_/*K*_m_ under this regime. The calculated value of *k*_1_ is 0.020 ± 0.002 min^–1^ × µM^–1^ (1.2 × 10^6^ s^–1^ × M^–1^) in good agreement with the rate constant reported for EcRNH1, which ranged from 0.58 × 10^6^ s^–1^ × M^–1^ to 6.5 × 10^6^ s^–1^ × M^–1^ depending on the R-loop structure [53]. Therefore, it could be concluded that the efficiency of R-loop degradation by both RNase H1s mostly depends on the rate of the catalytic complex’s formation and proceeds by the same mechanism (Scheme 1).

## 3. Discussion

This study provides the first comparative analysis of the R-loop degradation activity of the chloroplast-localized RNase H1 enzyme from *Arabidopsis thaliana*. The research demonstrates that recombinant AtRNH1C is an active enzyme that cleaves the RNA within DNA/RNA, with a distinct preference for purine-rich sequences—particularly at G↓X dinucleotides. This cleavage specificity aligns with previous observations for both *E. coli* and human RNase H1, suggesting an evolutionarily conserved catalytic mechanism across kingdoms. Kinetic analysis revealed that the enzyme’s efficiency is highly dependent on the length of the DNA/RNA hybrid region. The most efficiently processed substrate was R-loop11, which mimics the natural ~11 bp hybrid length maintained by RNA polymerase in transcription elongation complexes. The efficiency of RNA degradation decreased as the hybrid length increased (R-loop16, -21, and -31), indicating that AtRNH1C is exquisitely tuned to resolve the short, transient R-loops that occur co-transcriptionally, rather than processing excessively long, stable structures.

According to the proposed model for DNA/RNA hybrid degradation by human RNase H1 [47], the HBD enhances RNase H1′s affinity for DNA/RNA hybrids. In human RNase H1, the long linker (60 aa) appears to moderately enhance substrate binding (approximately 2.5-fold), but it also enables the catalytic domain to interact with the substrate at multiple sites, which may be important for increased processivity [47]. After hydrolysis, when the catalytic RNH1 domain separates from the cleavage product, the HBD may anchor the enzyme (or at least promote its reassociation) to the substrate, allowing another cleavage event nearby [47]. This processive behavior allows the enzyme to generate smaller nucleotide products compared to the catalytic domain acting alone. It is important to note that processivity was most evident during the degradation of long hybrids and had minimal impact on shorter substrates [46], as only minor differences in degradation product distribution were seen between *E. coli* RNase H1 and mouse RNase H1 [46]. In AtRNH1C, the flexible linker between the HBD and RNH1 domain is 111 aa long. It is reasonable to assume that its effect on substrate binding may also be moderate. We did not investigate the HBD’s influence on substrate binding because, despite extensive efforts, we could not produce a soluble recombinant protein of the truncated AtRNH1C without the HBD domain. The model R-loop structures we used contained a DNA/RNA hybrid no longer than 31 bp, so estimating processivity would be highly speculative. What are the possible lengths of R-loops? Studies indicate that R-loops can vary from a few base pairs to several hundred base pairs [12,55]. Although R-loop formation is typically associated with transcription [56], evidence shows that some R-loops can form independently of transcription, especially at DNA damage sites such as double-strand breaks [57], or through mechanisms related to DNA replication or the activity of certain non-B DNA structures and enzymes that can destabilize DNA duplexes. These transcription-independent pathways can still produce the characteristic DNA/RNA hybrid and displaced single-stranded DNA of R-loops, although they may also overlap or interact with transcription-dependent processes [5,58]. Clearly, further experiments are needed to directly test AtRNH1C processivity and the possible effects of the HBD on kinetics, processivity, or substrate preference.

The kinetic data support a two-step mechanism for AtRNH1C action, where the rate-limiting step is the formation of the initial catalytic complex (E•S). Thus, R-loop degradation by AtRNH1C is mostly dependent on the rate of catalytic complex formation and proceeds by the same mechanism as described for EcRNH1. Moreover, the rate constant of the catalytic complex formation for AtRNH1C is in good agreement with the rate constants reported for EcRNH1, suggesting a biologically conserved role of these enzymes in R-loop degradation. This remarkable conservation underscores a fundamental and shared enzymatic strategy for R-loop resolution, despite the phylogenetic distance between bacteria and plants. The presence of a G-quadruplex (G4) structure in the displaced DNA strand (R-loop31Q) did not significantly alter the cleavage rate, suggesting that AtRNH1C’s activity is primarily focused on the hybrid duplex itself and is robust against certain DNA secondary structures. The observed rate constant values of RNA cleavage by AtRNH1C in model R-loops show a drop in meaning between 11 and 16 nt followed by a plateau (Figure 5B). According to structural data, the catalytic domain of human RNase H1 interacts with 11 nt of the DNA/RNA hybrid [59]. It can be proposed that in the case of R-loop11, the higher efficacy of RNA cleavage reflected the effective binding of the DNA/RNA hybrid part by the RNH1 domain. The observed substrate preference of AtRNH1C, as well as the most efficient cleavage of the natural-like short R-loop11 generated in a transcription elongation complex, positions RNase H1 as one of important regulators of R-loop homeostasis in chloroplasts.

In a broader biological context, these findings position AtRNH1C as an important guardian of chloroplast genome integrity. Its optimized activity against short, transcription-associated R-loops suggests its role in preventing the accumulation of these structures, which could otherwise lead to transcription–replication conflicts, DNA damage, and impaired organelle function. By efficiently resolving R-loops, AtRNH1C collaborates with other enzymes like DNA gyrases to ensure the smooth progression of replication and transcription, thereby maintaining stability in the genetically vital chloroplast genome.

In summary, this work moves beyond genetic evidence and provides direct in vitro enzymatic validation of AtRNH1C’s function. It reveals the conserved kinetic and mechanistic principles that underpin R-loop degradation by RNase H1 enzymes and highlights the specific adaptation of AtRNH1C for maintaining homeostasis in the unique environment of the plant chloroplast. Taken together, this work elucidates the molecular mechanism of a plant organelle-specific RNase H1. Future investigations of the potential protein–protein interactions between AtRNH1C and other known chloroplast R-loop regulators, such as DNA gyrases or the helicase RHON1, as well as the comparison of the enzymatic properties of AtRNH1C with those of other RNase H1 isoforms (mitochondrial and nuclear) can provide the understanding of its functional specialization. For AtRNH1A and AtRNH1B, the canonical RNase H1 activity has been shown [37]; however, kinetic and mechanistic analysis was absent. Taking into account that the presence of AtRNH1B or AtRNH1C is required for viability, a direct kinetic comparison could provide a molecular basis for understanding the facultative dual targeting.

## 4. Materials and Methods

### 4.1. AtRNH1C Expression and Purification

The pGEX-4T-1-AtRNH1C plasmid encoding the wild-type of *A. thaliana* RNH1C was kindly provided by Prof. Qianwen Sun. AtRNH1C was produced in *E. coli* ArcticExpress (DE3) cells (1 L of 2YT medium with 20 µg/mL gentamicin, 10 µg/mL tetracycline, and 50 µg/mL ampicillin, shaking speed of 200 rpm) induced with 0.2 mM 1-thio-β-D-galactopyranoside (added after the optical density at 600 nm reached 0.6–0.7) at 37 °C for 3 h, providing recombinant protein expression in soluble form. The cells were then harvested (20 min at 5000× *g* at 4 °C) and suspended in 20 mM HEPES–KOH (pH 7.8), 140 mM NaCl, 5 mM DTT, and a mixture of protease inhibitors (Inhibitor cocktail, Complete, Mannheim, Germany). Cells were lysed using a French press and all subsequent procedures were performed at 4 °C. The lysate was filtered through a 0.45 µm MCE Syringe Filter (Labfil, ALWSCI Technologies Co., Hangzhou, China). The clarified lysate was incubated with 1 mL of GST-resin (Glutathione Sepharore^TM^ 4B, Uppsala, Sweden) at 4 °C for 1 h with gentle shaking. The resin was washed 3 times by 10 mL of 50 mM Tris–HCl (pH 8.0) with 140 mM NaCl and protein was eluted by 3 mL of 50 mM Tris–HCl (pH 8.0) with 140 mM NaCl and 10 mM reduced glutathione. Protein purity was examined using SDS–polyacrylamide gel electrophoresis. The qualitative assessment of protein purity showed the absence of contaminant proteins (Figure S1). The removal of the GST tag by thrombin resulted in protein precipitation. In order to exclude protein precipitation, the stage of GST tag cleavage was not carried out. The purified AtRNH1C was concentrated with a 30 kDa JetSpin Centrifugal Filter (Jet Bio–Filtration Co., Ltd., Guangzhou, China), and the resulting protein was stored at –20 °C in a buffer consisting of 25 mM Tris–HCl (pH 8.0), 70 mM NaCl, 5mM reduced glutathione, and 50% glycerol. The purified protein yield was 2.1 mg from a 1 L bacterial culture. The enzyme concentration was calculated from the optical density of the protein at 280 nm and a molar extinction coefficient of 68,480 M^−1^ × cm^−1^. All experiments were conducted in an autoclaved H_2_O treated with 0.1% DEPC (37 °C, 2 h) unless otherwise stated.

### 4.2. Preparation of R-Loops

The sequences of the model R-loops are shown in Figure 2. DNA oligonucleotides were purchased from Biosset Ltd. (Novosibirsk, Russia). RNA oligonucleotides with a 5′-FAM label were synthesized by the Laboratory of RNA Chemistry at ICBFM SB RAS (Novosibirsk, Russia), as previously described [60]. Oligonucleotide concentrations were determined by measuring absorbance at 260 nm, and the R-loops were assembled according to an established protocol [53]. Briefly, for R-loops11, -16, -21, and -31, non-template DNA strand, template DNA strand, and RNA primer at a 1:1:1 molar ratio were annealed in 1× buffer containing 20 mM HEPES-KOH (pH 7.2), 75 mM NaCl, 5 mM MgCl_2_, and 1 mM DTT. For R-loop31Q, non-template DNA strand, template DNA strand, and RNA primer at a 1:1:1 molar ratio were annealed in 1× buffer containing 10 mM Na_2_HPO_4_, 1.8 mM NaH_2_PO_4_ (pH 7.5), 1 mM EDTA, 1 mM DTT, and 140 mM KCl. The formation of R-loops was analyzed by an electrophoretic mobility shift assay (EMSA) following a previously established method [53]. The EMSA was conducted in a nondenaturing 10% polyacrylamide gel (75:1) using 0.5× TBE buffer supplemented with 5 mM MgCl_2_, at a constant current of 10 mA and a temperature of 4 °C (Figure S2). The annealed 10× R-loop solutions were stored at –20 °C.

### 4.3. Cleavage Site Analysis

Site and sequence preferences for cleavage by AtRNH1C were identified using R-loops (Figure 2). For RNases A and U2, used as control enzymes, the cleavage of single-stranded RNA primers was analyzed as these enzymes degrade single-stranded RNA. Partial hydrolysis of single-stranded RNA primers with RNase A was performed as follows: a 20 μL reaction mixture containing 1.0 μM substrate and 13 pM RNase A in buffer (50 mM Tris-HCl pH 8.5, 50 mM NaCl, 1 mM EDTA, 1 mM DTT, and 9% glycerol) was incubated at 25 °C for 1 min [61,62]. For RNase U2, a 20 μL reaction containing 1.0 μM substrate and 2.7 μM enzyme in buffer (50 mM AcNH_4_, 1 mM EDTA, 1 mM DTT, and 9% glycerol) was incubated at 25 °C for 5 min. For AtRNH1C, a 20 μL reaction containing 1.0 μM substrate and 2.0 μM enzyme in buffer (20 mM HEPES-KOH pH 7.2, 75 mM NaCl, 5 mM MgCl_2_, 1 mM DTT, and 5% glycerol) was incubated at 25 °C for 30 min. The reactions were stopped by addition of an equal volume of a solution containing 9 M urea, 25 mM EDTA, 0.1% of xylene cyanole, and 0.1% of bromophenol blue followed by incubation at 96 °C for 5 min. The products were analyzed on 15% denaturing TBE-polyacrylamide gels using a vertical Protean II xi unit (Bio-Rad Laboratories, Hercules, CA, USA) at 200–300 V and 45 °C. All data were obtained at three replicates. Gels were visualized with a VersaDoc gel-documenting system (Bio-Rad Laboratories, Hercules, CA, USA).

### 4.4. Activity Assay

The reaction was conducted at 25 °C in buffer containing 20 mM HEPES-KOH (pH 7.2), 75 mM NaCl, 5 mM MgCl_2_, 1 mM DTT, and 5% glycerol with various enzyme/substrate ratios (enzyme concentrations, 0.5–4 µM; R-loop concentration, 1 µM). The reaction was stopped at certain time intervals by addition of an equal volume of a solution containing 9 M urea, 25 mM EDTA, 0.1% of xylene cyanole, and 0.1% of bromophenol followed by incubation at 96 °C for 5 min. A no-enzyme control was included in the first lane of all gels. Reaction products were resolved on 15% denaturing TBE-PAGE gels (Bio-Rad Protean II xi unit, Bio–Rad Laboratories, Inc., Hercules, CA, USA) at 200–300 V and 45 °C and imaged using a VersaDoc system (Bio-Rad Laboratories, Hercules, CA, USA). Quantification was performed with the GelAnalyzer 23.1.1 (©Istvan Lazar Jr.), where the transformation degree was calculated as follows: (sum of cleavage fragment peaks)/(sum of product peaks + initial substrate peak). Data from three replicates (mean ± SD) were fitted in OriginPro 8.1 (OriginLab Corporation, Northampton, MA, USA) to the equation [Product] = A × (1 − exp(−*k*^obs^ × t)), where A is the amplitude, *k*^obs^ is the observed rate constant, and *t* is the reaction time. The observed rate constants (*k*^obs^) were then plotted against the enzyme concentration and fitted linearly (*k*^obs^ = *k*_1_ × [AtRNH1C]) [63].

## Data Availability

The original contributions presented in the study are included in the article; further inquiries can be directed to the corresponding authors.

## Supplementary Materials

The following supporting information can be downloaded at https://www.mdpi.com/article/10.3390/ijms262211125/s1.
