# Supplementary material for: Biochemical Characterization of R-Loop Degradation by Chloroplast-Localized RNase H1 from Arabidopsis thaliana
Source: Int J Mol Sci. 2025 Nov 17;26(22):11125. doi: 10.3390/ijms262211125 (PMC12652420; doi:10.3390/ijms262211125)
Supplement: Supplementary file 1 [file ijms-26-11125-s001.zip › ijms-3960230-supplementary.pdf]

Biochemical Characterization of R-Loop Degradation by Chloroplast-Localized RNase H1 from *Arabidopsis thaliana*

Anastasia A. Gavrilova, Aleksandra A. Kuznetsova, Darya S. Novopashina, Chengxia Zheng, Qianwen Sun and Nikita A. Kuznetsov

Supplementary Materials

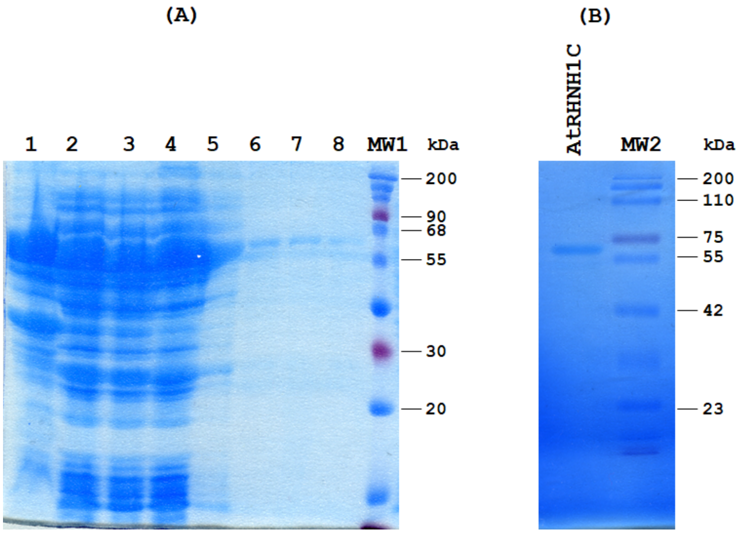

Figure S1. (A) Purification assay of AtRNH1C by SDS-PAAG. Probes: 1 – the cell pellet, 2 – the cell lysate obtained by the French-Press procedure, 3 – the cell lysate filtered through 0.45 µm MCE Syringe Filter, 4 – the flowthrough fractions after incubation with GST-resin, 5 – GST-resin washing without reduced glutathione, 6-8 – the protein fractions obtained by 10 mM reduced glutathione elution. (B) Analysis of the AtRNH1C purity after concentration with a 30 kDa JetSpin Centrifugal Filter (Jet Bio–Filtration Co., Ltd., China).

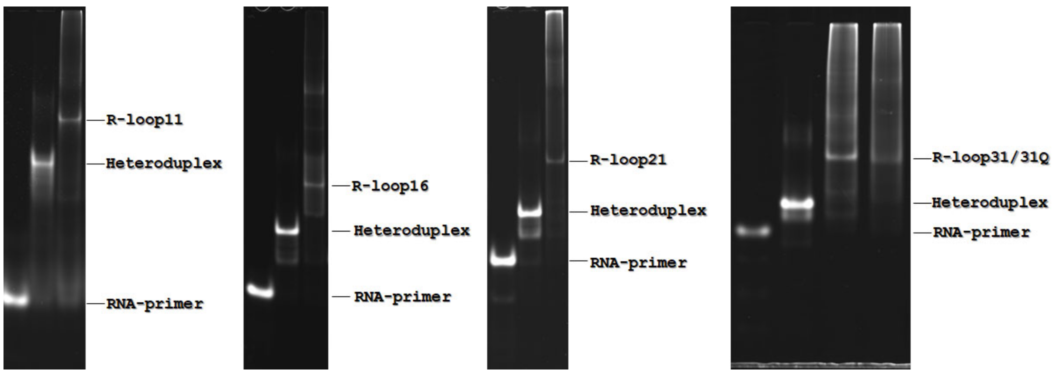

Figure S2. The characterization of R–loop formation using EMSA.
